# Effects of pre-sleep macronutrient ingestion on nocturnal and morning glycemic responses in elite female athletes: a pilot study

**DOI:** 10.1080/15502783.2025.2550208

**Published:** 2025-08-30

**Authors:** Sadio P. Fenner, Timothy D. Griest, Josh I. Murry, Matthew D. Vukovich, Michael J. Ormsbee

**Affiliations:** a Florida State University, Department of Health, Nutrition, and Food Sciences, Tallahassee, FL, USA; b Florida State University, Institute of Sports Sciences & Medicine, Tallahassee, FL, USA; c University of KwaZulu-Natal, School of Health Sciences, Discipline of Biokinetics, Exercise and Leisure Sciences, Durban, South Africa; d South Dakota State University, College of Education and Human Sciences, Vermillion, SD, USA

**Keywords:** Nutrient timing, sleep quality, glucose, athletes

## Abstract

**Background:**

Nutrient timing strategies are commonly employed by athletes to support recovery, sleep quality, muscle protein synthesis, and overnight metabolic regulation. However, limited research has explored the glycemic impact of different macronutrients consumed prior to sleep, particularly in elite female athletes. α-lactalbumin (ALA), a whey-derived protein rich in tryptophan, has been proposed to support stable overnight glucose levels and sleep quality. In contrast, carbohydrate (CHO) ingestion before bed is known to acutely elevate blood glucose, yet its influence on nocturnal glycemia during sleep remains less understood in high-performing athletes. This This study compared the effects of pre-sleep ingestion of ALA, casein (CAS), CHO, and a non-caloric placebo (PLA) on nocturnal continuous glucose monitoring (nCGM) metrics over a 4-week, randomized, double-blind, crossover study in elite female athletes.

**Methods:**

Each participant consumed one of four pre-sleep treatments—40 g of ALA, CAS, CHO, or a non-caloric PLA – for three consecutive nights per condition. Supplement was taken 2 hours after the final meal and 30 minutes before bedtime. Blood glucose was tracked every 15 minutes using continuous glucose monitoring (CGM) devices that were worn on the back of the arm for the duration of the study. Glycemic responses were collected for 24 hours each day of the study; starting from the two hours before the participants reported their bedtime, while they were sleeping, and continued up to the hour after the participant reported waking up was analyzed. Participants reported their bed and wake times in daily surveys, and CGM data was matched accordingly. Repeated measures ANOVA was used to evaluate the effects of time, condition, and time × condition interaction on glucose concentrations.

**Results:**

Six NCAA Division I female athletes (*n* = 6; Age: 22.5 ± 0.96 yrs, Height: 1.68 ± 0.06 m, Weight: 60.77 ± 7.02 kg) completed the study. A significant main effect of time on nocturnal glucose was observed (*p* = 0.008), reflecting expected glycemic variation during sleep. However, no significant differences were found between macronutrient conditions (*p* = 0.187), and there was no time × condition interaction (*p* = 0.550), suggesting the type of macronutrient ingested before bed did not significantly influence overnight glucose dynamics. Wake-time blood glucose values also did not differ significantly across conditions (*p* = 0.58).

**Conclusion:**

Pre-sleep ingestion of protein (ALA or CAS), carbohydrate, or placebo does not significantly impact nocturnal or next-morning glycemic control in elite female athletes. These findings suggest that pre-sleep macronutrient intake can be flexibly applied without negatively affecting overnight glucose regulation in elite female athletes.

